# Bacteriocinogenic potential and virulence traits of *Enterococcus faecium* and *E. faecalis* isolated from human milk

**Published:** 2017-08

**Authors:** Soodabeh Khalkhali, Naheed Mojgani

**Affiliations:** 1 Department of Microbiology, Shiraz Branch, Islamic Azad University, Shiraz, Iran; 2 Department of Microbiology, Fars Research and Sciences Branch, Islamic Azad University, Marvdasht, Iran; 3 Biotechnology Department, Razi Vaccine and Serum Research Institute, Agriculture Research Education and Extension Organization, Karaj, Iran

**Keywords:** *Enterococcus*, Bacteriocins, Biogenic amines, Human milk, Vancomycin, Virulence genes

## Abstract

**Background and Objectives:**

Human milk is a continuous supply of Lactic Acid bacteria (LAB), including enterococci with probiotic potentials. The aim of this study was to analyze two *Enterococcus* species, isolated from human milk for their probiotic potential, bacteriocin producing ability and virulence traits.

**Materials and Methods:**

*Enterococcus faecium* TA0033 and *E. faecalis* TA102 were tested for acid and bile tolerance, survival in simulated gastric and intestinal conditions. The antibacterial spectrum of the isolates was tested by agar well diffusion assay. The antagonistic agent was characterized by physico-chemical methods. The enterocin structural genes, virulence determinants, vancomycin resistance and biogenic amine genes, such as *hdc1*, *hdc2*, *tdc*, *ldc* and *odc* were also determined.

**Results:**

The tested isolates survived acidic conditions, high bile salt (1%), simulated gastric and intestinal conditions. The culture supernatant fluids of the two isolates inhibited the growth of *Escherichia coli*, *Listeria monocytogenes*, *Salmonella typhi*, *Staphylococcus aureus*, *Shigella dysenteriae* and *Streptococcus agalactiae*. The antagonistic activity was lost in the presence of proteolytic enzymes but tolerated the action of catalase, lysozyme and lipase. In contrast to enterocin TA102, enterocin TA0033 possessed bactericidal mode of action. Bacteriocin structural genes, *entA* and *entB* were present in the genome of the two isolates, while *E. faecalis* TA102 additionally harboured *entP* and *bac31* genes. The phenotypic and genotypic virulence assessment studies indicated hyaluronidase (*hyl*) production and vancomycin resistance in *E. faecalis* TA102 while, none of the isolates harboured the biogenic amine genes.

**Conclusion:**

The presence of virulence genes in *E. faecalis* TA102 calls for careful monitoring of *Enterococcus* isolates for their safety parameters.

## INTRODUCTION

Enterococci are Gram-positive cocci well known for their role in enhancing the shelf life, developing flavor compounds in food products, improving the intestinal microbial balance and treating gastroenteritis in humans and animals (1). Owing to the known health benefits of these bacteria they are used in a number of probiotic formulations. A significant character of *Enterococcus* species is their bacteriocin producing ability, which is a useful biotechnological trait (2). To date, a number of bacteriocin producing enterococci has been isolated from breast milk, and their bacteriocins (enterocins) been characterized (3). Enterocins are defined as small, ribosomally synthesized peptides displaying inhibitory actions, against the target bacteria by either dissipation of proton motive force by pore formation, cell lysis, or interference with degradation and metabolism of macromolecules (4). A number of reports have indicated the presence of virulence traits in this group of bacteria and their safety aspects are still under considerations. All this calls for strict scrutinzations before considering them as a suitable probiotic candidate. Some of the significant virulence genes reported to date are; *ace* (collagen binding protein), *asa1* (aggregation substance), *cpd* (sex pheromone peptides), *cylA* (cytolysin activation), *cylB* (cytolysin transport), *cylM* (posttranslational modification of cytolysin), *efaA* (endocarditis antigen), *esp* (enterococcal surface protein), *gelE* (Gelatinase), and *hyl* (hyaluronidase) (5).

In the last few decades, vancomycin resistant Enterococci have emerged, causing major problems in infection control. Three phenotypic classes (VanA, VanB and VanC) are defined by the level of resistance to vancomycin and susceptibility or resistance to teicoplanin (6). Moreover, biogenic amines (BA) present in food are known to have deleterious health effects on humans, and thus selection of strains with no BA producing ability is highly recommended (7, 8). Hence, before we could exploit these bacteria in the food or biopharmaceutical industry, and or as a probiotic supplement, it is essential to evaluate their safety. In this study, we evaluated the bacteriocinogenic and virulence characteristics of two *Enterococcus* isolates by phenotypic and genotypic methods.

## MATERIALS AND METHODS

### Bacterial strains and cultivation conditions.

*E. faecium* TA0033 and *E. faecalis* TA102, isolated from human milk samples of healthy young mothers in Tehran, capital of Iran, were identified by their carbohydrate fermentation pattern and 16S rRNA sequencing (9, 10). The isolates were cultured on KF agar, supplemented with 1% TTC (2, 3, 5-Triphenyl-Tetrazolium Chloride Solution, SIGMA, UK) and Kanamycin aesculin-Azide Agar (KAA, Oxoid, UK). All other Gram-positive and Gram-negative pathogens, used in the study were grown in BHI (Brain heart Infusion, Merck, USA) broth, at 37°C for 24 h in aerobic conditions. Stock-cultures were maintained in MRS broth supplemented with 20% glycerol and stored at −70°C.

### Probiotic characterization of the isolates: Acid and bile tolerance.

The isolates were screened for their acid and bile resistance, by culturing in MRS broth with different set pH values (2, 2.5, 3, 4, 5, 6) and bile concentrations (0.1, 0.5, 0.7, 1%). The resistance of the isolates at the tested pH values was recorded, by determining their growth (cfu/ml) at different time intervals. While, bile tolerance was estimated, by calculating the Coefficient of inhibition (C _inh_ ), according to the formula described by Gopal et al. (11).

$${\text{C}}_{\text{inh}}=\Delta_{\text{T}8-\text{T}0}\text{Control}-\Delta_{\text{T}8-\text{T}0}\text{Treatment}/\Delta_{\text{T}8-\text{T}0}\text{Control}$$

Where, Δ _T8-T0_ represents the difference in absorbance at time zero (T0) and after 8 h (T8). C _inh_ of less than 0.4 was considered significant, for the isolates to be considered as a suitable probiotic candidate.

### Survival in simulated gastric and intestinal conditions.

The survival of the selected isolates under simulated gastric and intestinal contents was studied, by previously described method (12). The bacterial survival under the tested conditions was calculated as:

R=Average of cells at 10min/Average of cells at 0min

According to the formula, R =1 when no effect on the growth and survival of bacteria is seen, while a ratio of 0.5, indicated a loss of 50% of the viability. Ratios greater than 1 indicated bacterial growth.

### Antibacterial spectrum.

The inhibitory spectrum of the selected isolates was studied, by determining the antagonistic action of their neutralized supernatant fluids (NSF), against a number of pathogens by agar well diffusion assay (13). Freshly grown cultures of a number of Gram-positive and Gram-negative pathogens, listed in Table 3, were used as sensitive indicator cells. Zone diameters, surrounding the wells were measured in millimeters, and based on these results the producer isolates were recorded as strong (≥20mm), moderate (≥16–19 mm) and weak (≤15 mm). The antibacterial activity demonstrated by the isolates was quantified, by slight modifications in the critical dilution method, described by Schillinger and Lucke (13). Two fold serial dilutions (100 μl) of NSF were poured in wells in agar plates seeded with the indicator strain, and incubated aerobically at 37ºC for 24 h. The diameters of the inhibition zones were measured in millimeters, by subtracting the well diameter from the zone diameter. The results were expressed in arbitrary units per millimeter (AU/ml), defined as the reciprocal of the highest dilution demonstrating zone of inhibition.

**Table 3.** Effect of heat treatment on the enterocin activity (AU/ml) of the *Enterococcus* isolates, at different time intervals

**Time (min)**	***Enterocin* TA0033 (AU/ml)**				***Enterocin* TA102 (AU/ml)**								

	**60°C**	**80°C**	**100°C**	**120°C**	**60°C**	**80°C**	**100°C**	**120°C**
0	25600	25600	25600	25600	6400	6400	6400	6400
15	25600	25600	0	0	6400	6400	6400	6400
30	12800	6400	0	ND	6400	6400	6400	0
60	6400	6400	0	ND	6400	6400	1600	ND
90	6400	1600	0	ND	6400	6400	0	ND

ND: not determined

### Physicochemical characterization of the antagonistic agents.

The antagonistic agents produced by the two isolates were physico-chemically characterized. The effect of H _2_ O _2_ for the antimicrobial activity was excluded, by determining the antagonistic activity in the NSF after subjection to the enzyme catalase (1 mg/l). While, the chemical nature of the antagonistic agent was analyzed by treating the NSF of the producer strains with the enzymes like lipase, lysozyme, pepsin, pronase E, and proteinase K (Fluka, England), at a final concentration of 1 mg/l in phosphate buffer (10, 14).

Effect of variable pH (2.0, 4.0, 6.0, 8.0, and 10.0) and temperature ranges (60, 80, 100, 121°C) on the NSF of the two producer isolates, at different time intervals of 15, 30, 60, 90 min, was studied.

The bactericidal or bacteriostatic activity of the studied bacteriocins was determined by critical dilution assay using *S. aureus* as indicator strain. After 24 h, 5 μl of 10 mg/ml proteinase K solution (Sigma, USA) were spotted near each inhibition zone and the plates were again incubated at 37°C and observed for presence or absence of growth of indicator strain. Bactericidal mode of action of the bacteriocins was determined by the absence of indicator strain growth, after the destruction of the inhibitor by proteases. Absence of inhibition zones after enzymatic treatment indicated a bacteriostatic mode of action.

### Enterocin structural genes.

The presence of bacteriocin structural genes in the producer isolates was studied, using a set of primers in a PCR assay. Multiple pairs of enterocin structural genes were used, as described previously (14, 15). DNA template was prepared by suspending a loop full of bacterial colony in 10μl of lysis buffer (0.25% SDS/ 0.05 % NaOH), heated at 95°C for 5 min and centrifuged at 15 000 × g for 5 min. The samples were diluted in 90 μl of sterile distilled water, centrifuged as above and the supernatant used as template DNA. Cycling parameters included a 2 min initial denaturation at 94°C, followed by 40 cycles of 45 s at 95°C, 30 s at 56°C for *entP*, *bac31* and *entL50A/B*, 58°C in the case of *entA* and 60°C for *entB*, *entQ*, and *cyl* as annealing temperature, and 60 s at 72°C. Amplified PCR fragments were resolved on 1% agarose gels, using a 100 bp ladder for size verification.

### Biochemical virulence traits.

The two isolates were screened for hemolytic activity, arginine hydrolysis, gelatinase, lipase, DNase, lecithinase and hyaluronidase production (16).

The ability of the mentioned *Enterococcus* isolates to produce biogenic amines (tyramine, histamine, putrescine, and cadaverine) was determined, using the decarboxylase broth and the method described by Bover-Cid and Holzapfel (17).

The phenotypic resistance of the selected isolates to vancomycin (30ug, Sigma, USA) was determined, by disc diffusion assay reported previously (18). According to the recommendation of the Clinical and Laboratory Standards Institute (CLSI), the strain was considered to be resistant to antibiotics, if the inhibition zone was ≤ 14 mm for the indicated antibiotic.

### Genotypic virulence determinants.

Virulence in *Enterococcus* species was determined by PCR assay, using a set or primers targeting the virulence genes. Primer sequences and expected amplicons size are listed in Table 1. *E. faecalis* ATCC 29212; ATCC 51299 and *E. faecium* ATCC-BAA 2320; ATCC 19434 were used as positive control during this study. PCR parameters used, were according to the respective references (Table 1).

**Table 1.** Virulence genes and their sequences, used in the study

**Genes**	**Primer Sequences (5-3)**	**Amplicon size (bp)**	**Reference**
**Virulence genes**			
*agg-F*	AAGAAAAAGAAGTAGAGACCAAC	1553	(1)
*agg-R*	AAACGGCAAGACAAGTAAATA		
*asa1-F*	GCACGCTATTACGAACTATGA	375	(19)
*asa1-R*	TAAGAAAGAACATCACCACGA		
*cpd-F*	TGGTGGGTTATTTTTCAATTC	782	(19)
*cpd-R*	TACGGACTCTGGCTTACTA		
*cylA-F*	ACTCGGGGATTGATAGGC	688	(19)
*cylA-R*	GCTGCTAAAGCTGCGCTT		
*cylB-F*	AAGTACACTAGTAGAACTAAGGGA	2020	(19)
*cylB-R*	ACAGTGAACGATATAACTCGCTATT		
*cylM-F*	AAAAGGAGTGCTTACACTGGAAGAT	2940	(19)
*cylM-R*	CATAACCCACACCACTGATTCC		
*efaA-F*	GACAGACCCTCACGAATA	705	(1)
*efaA-R*	AGTTCATCATGCTGTAGTA		
*esp-F*	AGATTTCATCTTTGATTCTTGG	510	(19)
*esp-R*	AATTGATTCTTTAGCATCTGG		
*gelE-F*	TATGACAATGCTTTTTGGGAT	213	(19)
*gel-R*	AGATGCACCCGAAATAATATA		
*hyln-F*	ACAGAAGAGCTGCAGGAAATG	276	(19)
*hyln-R*	GACTGACGTCCAAGTTTCCAA		
**Vancomycin resistance genes**			
VanA*-F*	CGGGGAAGATGGCAGTAT	732	(22)
VanA*-R*	CGCAGGGACGGTGATTTT		
VanB*-F*	CGGGGAAGATGGCAGTAT	635	(22)
VanB*-R*	CGCAGGGACGGTGATTTT		
VanC*-F*	CGGGGAAGATGGCAGTAT	484	(22)
VanC*-R*	CGCAGGGACGGTGATTTT		
**Genes for biogenic amines**			
hdc1-F	AGATGGTATTGTTTCTTATG	367	(20)
hdc1-R	AGACCATACACCATAACCTT		
hdc2-F	AAYTCNTTYGAYTTYGARAARGARG	534	(20)
hdc2-R	ATNGGNGANCCDATCATYTTRTGNCC		
tdc-F	ACATAGTCAACCATRTTGAA	1100	(21)
tdc-R	CAAATGGAAGAAGAAGTAGG		
ldc1-F	TTYGAYWCNGCNTGGGTNCCNTAAC	1098	(20)
ldc1-R	CCRTGDATRTCNGTYTCRAANCCN		
odc-F	TGCACTTCCATATCCTCCAG	127	(23)
odc-R	GAATTTCTGGAGCAAATC		

In order to determine Vancomycin resistance genes in the test isolates, three sets of primers namely VanA, VanB and VanC were used in a PCR reaction, as described earlier (19).

The primer sequences targeting genes for amino acid decarboxylases, including *hdc1* and *hdc2* (both related to histidine decarboxylase), *tdc* (tyrosine decarboxylase), *ldc* (lysine decarboxylase) and *odc* (ornithine decarboxylase) were selected for studies. Primer sequences and PCR parameters were, as described previously (1, 6, 19–23).

### Statistical Analyses.

Differences in the prevalence of virulence genes between the two *Enterococcus* species were compared, using the Chi-square test with a p-value < 0.05, indicating statistical significance.

## RESULTS

*E. faecium* TA0033 and *E. faecalis* TA102 isolated from colostrum of healthy mothers in a previous study (NCBI Gene Bank with accession numbers KX158836.1 and KY009901.1, respectively), were evaluated for their probiotic potential and safety traits.

**Fig. 1.**
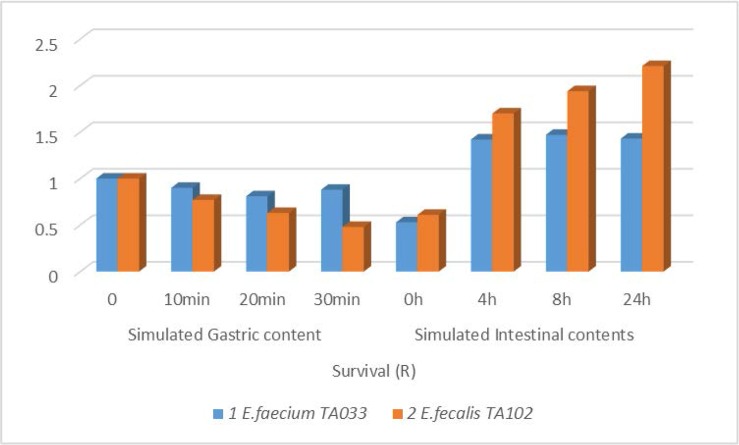
The survival rate of *Enterococcus* isolates in stimulated gastric and upper intestine contents, at different time intervals

*In vitro* probiotic characterization of the isolates indicated the isolates to be resistant to acidic pH values of 2.5 and above, while none could survive lower pH values of 2. Enhanced survival rate of the tested *Enterococcus* isolates in the presence of 1% bile concentrations was observed, as their coefficient of inhibition (C _inh_ ) values, appeared to be less than 0.4%. The survival rate of the selected isolates, in simulated gastric and intestinal content was indicated by their calculated R value. Fig. 1 shows survival of *E. faecalis* TA102 in simulated gastric conditions to be below 50 %, while in simulated intestinal conditions their survival percentage was significantly higher (R value > 2), compared to *E. faecium* TA0033.

The cell free supernatant fluid (CFSF) of the *Enterococcus* isolates demonstrated wide antibacterial spectrum, against a range of Gram-positive and Gam-negative pathogens. The CFSF of both the isolates were inhibitory towards the growth of *E. faecium*, *E. coli*, *L. monocytogenes*, *S. typhi*, *S. aureus*, *Sh. dysenteriae* and *S. agalactiae*. However, none of the isolates could inhibit the growth of *S. pyogenes* (Table 2). The spectrum of activity of *E. faecium* TA0033 appeared significantly higher than *E. faecalis* TA102 (P≤0.05).

**Table 2.** Antibacterial spectrum of cell free supernatant fluids of *Enterococcus* species, against pathogens

**Bacterial Pathogens**	**Reference Type**	***E. faecium* TA0033**	***E. faecalis* TA102**
*Bacillus cereus*	PTCC 1015	W	N
*Bacillus subtilis*	RTCC 1058	S	M
*Enterococcus faecium*	ATCC 19434	S	S
*Enterococcus faecium*	ATCC-BAA2320	W	N
*Enterococcus faecalis*	ATCC 29212	S	W
*Enterococcus faecalis*	ATCC 51299	W	N
*Escherichia coli*	RTCC 1162	S	S
*Escherichia coli O157:H7*	ATCC 43888	N	S
*Escherichia coli K99*	Unknown	W	N
*Klebsiella pneumoniaae*	PTCC1290	W	N
*Listeria ivanovii*	RTCC 13311	N	M
*Listeria monocytogenes*	RTCC 1240	M	S
*Pasteurella multocida*	ATCC 43137	W	N
*Pseudomonas aeruginosa*	RTCC 1502	S	M
*Salmonella typhi*	Local isolate	S	S
*Shigella dysenteriae*	PTCC 1188	W	S
*Staphylococcus aureus*	ATCC 64542	S	S
*Staphylococcus epidermidis*	ATCC 12228	N	W
*Streptococcus agalactiae*	RTCC 2051	S	S
*Streptococcus pyogenes*	ATCC 19615	N	N

ATCC: American type culture collection; PTCC: Persian type culture collection; RTCC: Razi type culture collection.

S: Strong anti-bacterial activity (zone diameter ≥20mm)

M: Moderate anti-bacterial activity (zone diameter ≥16–19mm)

W: Weak anti-bacterial activity (zone diameter ≤15mm)

N: No anti-bacterial activity (absence of zone of inhibition)

All experiments performed in triplicate.

**Fig. 2.**
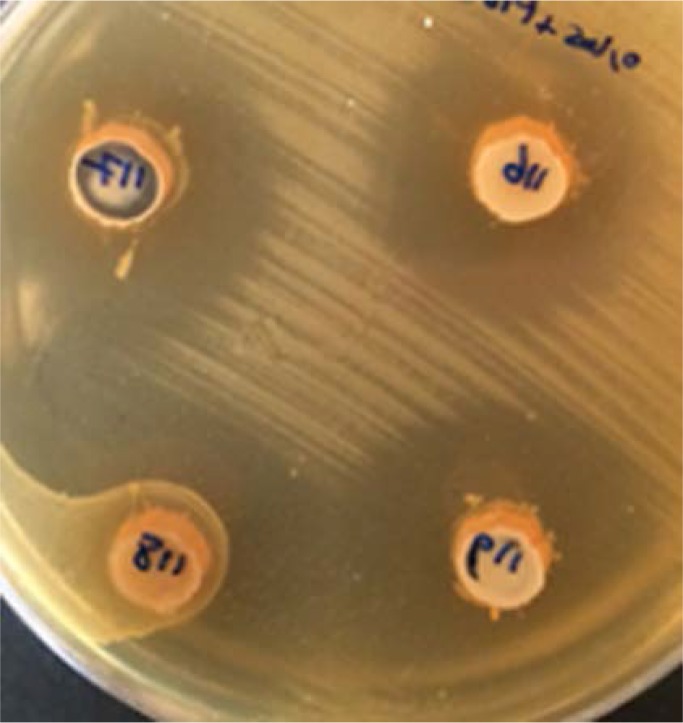
Effect of neutral pH, catalase, lipase and lysozyme on the enterocin produced by *E. faecium* TA0033

The inhibitory activity demonstrated by the isolates, appeared unaffected by neutralization of their supernatant fluid and the action of enzyme catalase. These results indicated the absence of acid and H _2_ O _2_ for the anti-bacterial activity ( Fig. 2). The proteolytic nature of the antagonistic agent produced by both the isolates was confirmed, by their sensitivity to the tested proteolytic enzymes. In contrast, lipase and lysozyme had no effect on the inhibitory actions of the two isolates.

**Fig. 3.**
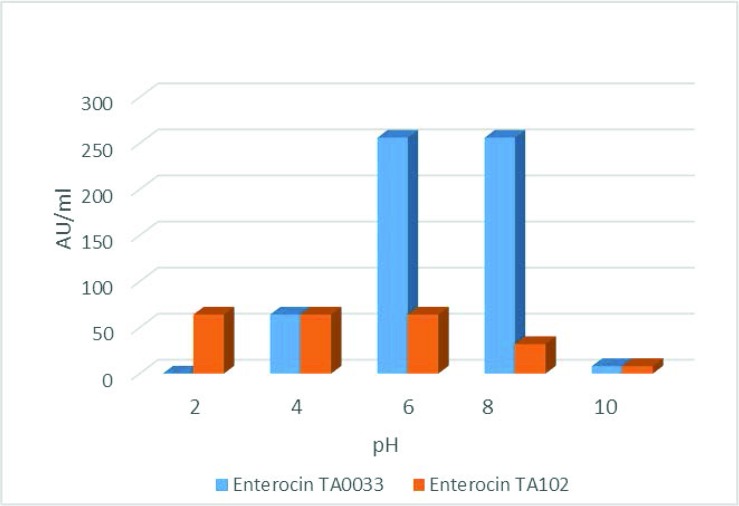
Acid resistance of enterocin TA0033 and enterocin TA102 at different pH within 24 h.

Effect of variable pH on the proteinaceous antagonistic compound produced, by the *Enterococcus* isolates in study is illustrated in Fig. 3 Significant differences were recorded in the pH stability of the studied enterocins (P≤0.05). According to the obtained results, enterocins TA0033 was not able to resist the extreme acidic (pH 2.0) and alkaline (pH 10) conditions, whereas, enterocin TA102 retained its stability at pH 2.0 for 24 h. Both the enterocins lost their activity completely, at an alkaline pH 10 and above. Maximum activity (AU/ml) was recorded at pH values of 2.0, 6.0 and 8.0 in both the isolates.

Thermal stability of enterocins TA0033 and TA102 is depicted in Table 3. Significant differences (p≤0.05) were recorded, in the thermal stability of the two enterocins. Enterocin TA102 appeared to be highly temperature resistant, compared to enterocins TA0033, as it was able to resist 100°C for 60 min and 121°C for 15 minutes.

In contrast, enterocin TA0033 was unstable at 100°C and autoclaving temperatures and lost its activity after exposure to the indicated temperatures. Partial loss in activity was seen with enterocin TA0033 at 60 and 80°C after 60 min of exposure with 75% reduction in activity within 90 min. In contrast, no loss of activity of enterocin TA102 was seen at these temperatures, during the tested time intervals and no significant difference (p>0.05), in activity between treated and untreated supernatant were observed.

Enterocin TA0033 displayed bactericidal mode of action at 6, 400 AU/ml, whereas bacteriostatic activity was detected at lower concentrations (200 AU/ml). Enterocin TA102 demonstrated bacteriostatic mode of action, as seen by the absence of inhibition zones after enzymatic treatment.

Multiple enterocin genes were detected in the tested isolates. The isolates possessed *entA* and *entB*, as indicated by a visible band of 126 and 159 bp, respectively. Whereas, *entP* and *bac31*, corresponding to DNA bond size of 121 bp and 248 bp, respectively, were detected only in *E. faecalis* TA102.

**Fig. 4.**
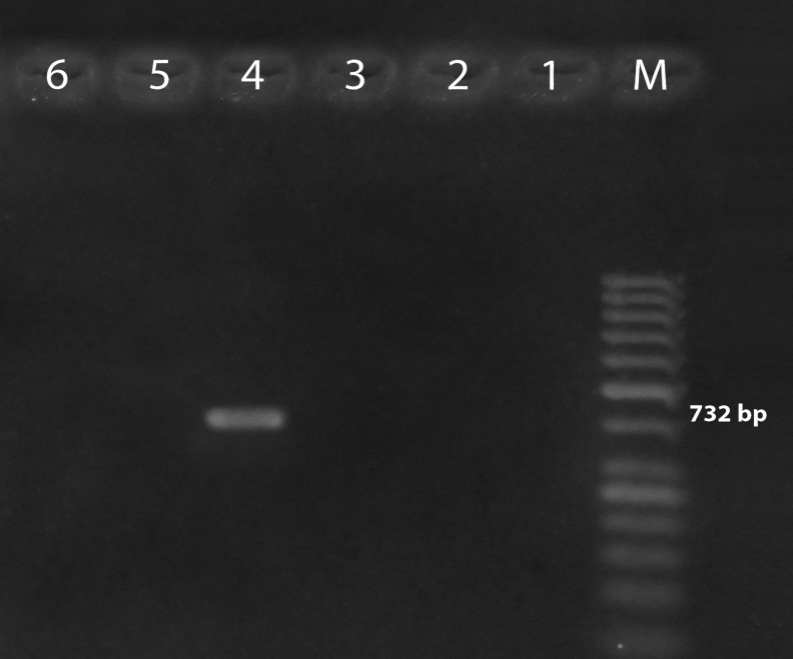
Vancomycin resistance genes in *E. faecium* TA033 and *E. faecalis* TA102. Lanes M: Molecular weight marker; Lane 1, 2, 3: vanA, vanB and vanC in *E. faecium* TA0033; Lanes 4, 5, 6: vanA, vanB and vanC in *E. faecalis* TA102

The phenotypic and genotypic virulence traits of the isolates are indicated in Table 4. During virulence trait characterizations, *E. faecium* TA0033 appeared non virulent as none of the tested virulence factors were observed in this isolate during biochemical and molecular genetic analysis. However, *E. faecalis* TA102 showed the presence of *cylA* (688 bp, cytolysin), *esp* (510bp, Enterococcal surface protein) and vanA (732 bp, Vancomycin) (Fig. 4).

**Table 4.** Phenotypic and genotypic virulent characters in *Enterococcus* isolates

**Virulence determinants**	***E. faecium* TA0033**	***E. faecalis* TA102**
**Phenotypic virulence traits**		
Hemolytic activity	α	γ
Arginine hydrolysis	−ve	−ve
Gelatinase production	−ve	−ve
Lipase production	−ve	−ve
DNase production	−ve	−ve
Lecithinase production	−ve	−ve
Hyaluronidase production	−ve	−ve
Vancomycin (30ug)	S	R
**Genotypic Virulence traits**		
*agg*	−ve	−ve
*asa*	−ve	−ve
*cpd*	−ve	−ve
*cylA*	−ve	+ve
*cylB*	−ve	−ve
*cylM*	−ve	−ve
*efa*	−ve	−ve
*esp*	−ve	+ve
*gelE*	−ve	−ve
*hyl*	−ve	−ve
**Genes for Vancomycin Resistance**		
vanA	−ve	+ve
vanB	−ve	−ve
vanC	−ve	−ve
**Genes for Biogenic Amines**		
*hdc1*	−ve	−ve
*hdc2*	−ve	−ve
*tdc*	−ve	−ve
*ldc*	−ve	−ve
*odc*	−ve	−ve

The genomic DNA of the two Enterococcal isolates was subjected to the genes coding for the enzymes involved in biogenic amines (BA) production, including *hdc1*, *hdc2*, *tdc*, *ldc*, and *odc*. None of the respective genes appeared to be present in the genome of the tested *Enterococcus* species and the two isolates were considered BA negative.

## DISCUSSION

Human milk is a potential source of probiotic bacteria, including lactobacilli, streptococci, bifidobacteria and enterococci to the infant gut, affecting the overall composition of the neonate gut microbiota. Although enterococci are widely recognized as probiotic bacteria, but opposed to other LAB genus they have yet not been assigned the GRAS (generally recognized as safe) status. *E. faecium* is considered a suitable probiotic candidate for the modulation of immune responses against pathogens (24). In this study, we observed the probiotic properties of the two *Enterococcus* species, including their resistance in stress conditions like acidic environment, high bile salt concentrations, and simulated gastric and intestinal conditions. Another important characteristic of the isolates in the study was their wide antibacterial spectrum against a number of Gram-positive and Gram-negative pathogens. In agreement with our findings, Ghrairi and colleagues (25), reported *E. faecium* MMT21 bacteriocin ability to inhibit not only closely related LAB, but also *L. monocytogenes* and *S. aureus*. Different spectrum of inhibitory action may be based on the bacteriocin producing strain, the indicator strain, and also the method used for bacteriocin detection. During physicochemical characterizations of the antagonistic agent produced by the two *Enterococcus* species in study, it was observed that the antibacterial actions exerted by the isolates were not due to acids or hydrogen peroxide. Similar to other reports (26), enterocin TA0033 and TA102 appeared to be simple proteins rather than conjugated proteins linked to lipid or carbohydrate moiety. Temperature and pH stability of enterocins is considered an essential aspect, which may compromise their use in dairy products (27). Comparable to enterocin SE-K4, enterocin CRL 1826, mundticin KS and enterocin QU2 (28), Enterocin TA102 showed significant pH and thermal stability. The mentioned enterocin TA102, showed significantly higher resistance in these conditions, compared to other reported enterocins produced by *E. faecium* D081821, and *E. faecium* D081833.

Presence of more than one bacteriocin gene in *Enterococcus* species has been reported previously ( 4, 10, 12, 14). In agreement with these reports, we observed multiple enterocins genes in both of the producer isolates. Nevertheless, the incidence of numerous enterocin genes in enterococci is not necessarily associated with an enhanced bacteriocin activity, and not all enterocin genes should be expressed at the same time (29).

A microorganism considered, as a probiotic is essentially either of GRAS status or their safety parameters are required to be investigated, before they could be considered a probiotic. *Enterococcus* species have been known to be responsible for nosocomial infections, especially in neonates, and those who suffer from underlying diseases (30). Therefore, the enterococcal strain of clinical or industrial interest should be carefully and individually evaluated for their safety and associated risk factors. In this study, we could demonstrate the safety of *E. faceium* TA0033, as none of the tested phenotypic or genotypic virulent traits were observed in this isolate. In contrast, *E. faecalis* TA102 showed the presence of few pathogenic genes like *cylA* and *esp*. Our results are in accordance with the previous findings, stating that pathogenic virulence factors are more common in *E. faecalis* strains compared to *E. faecium* strains (31).

Antibiotic resistance is one of the major safety concerns in Enterococci strains, particularly vancomycin resistance, as it is the drug of choice efficient against clinical infections by multidrug resistance pathogens. In our studies, *E. faecalis* TA102 appeared vancomycin resistant, during phenotypic and genotypic analysis. According to the reports, if the strain has either of the vancomycin resistance genes such as vanA, vanB and vanC, it is considered unsafe and could not be applied in the food or feeds (32). Another important safety concerns, are biogenic amine production by some probiotic isolates to be used in the dairy industry. Biogenic amines contained foods are known to have toxicological properties and are known to trigger health problems such as allergies, hypertension, hypotension, headaches, depressions, schizophrenia and Parkinson disorder (7) and thus should be avoided in food (33, 34). Absence of BA genes highlights the importance of a strain for use in starter and adjunct cultures.

In conclusion, results of this study determine the possible advantage of the indicated enterocins in dairy or biotechnology industry. The presence of couple of virulence genes in *E. faecalis* TA102 calls for careful monitoring of *Enterococcus* isolates, for their safety parameters.
